# Consequences of Aberrant Insulin Regulation in the Brain: Can Treating Diabetes be Effective for Alzheimer’s Disease

**DOI:** 10.2174/157015911798376334

**Published:** 2011-12

**Authors:** L Arab, R Sadeghi, D.G Walker, L-F Lue, M.N Sabbagh

**Affiliations:** 1 The Cleo Roberts Center for Clinical Research, Banner Sun Health Research Institute, Sun City, Arizona, USA; 2 Midwestern University School of Osteopathic Medicine, Glendale, Arizona, USA; 3 Laboratory for Neuroinflammation, Banner Sun Health Research Institute; 4 Laboratory for Neuroregeneration, Banner Sun Health Research Institute, Sun City, Arizona, USA

**Keywords:** Alzheimer’s disease, pathology, neurodegeneration, glucose metabolism, amyloid beta.

## Abstract

There is an urgent need for new ways to treat Alzheimer’s disease (AD), the most common cause of dementia in the elderly. Current therapies are modestly effective at treating the symptoms, and do not significantly alter the course of the disease. Over the years, a range of epidemiological and experimental studies have demonstrated interactions between diabetes mellitus and AD. As both diseases are leading causes of morbidity and mortality in the elderly and are frequent co-morbid conditions, it has raised the possibility that treating diabetes might be effective in slowing AD. This is currently being attempted with drugs such as the insulin sensitizer rosiglitazone. These two diseases share many clinical and biochemical features, such as elevated oxidative stress, vascular dysfunction, amyloidogenesis and impaired glucose metabolism suggesting common pathogenic mechanisms. The main thrust of this review will be to explore the evidence from a pathological point of view to determine whether diabetes can cause or exacerbate AD. This was supported by a number of animal models of AD that have been shown to have enhanced pathology when diabetic conditions were induced. The one drawback in linking diabetes and insulin to AD has been the postmortem studies of diabetic brains demonstrating that AD pathology was not increased; in fact decreased pathology has often been reported. In addition, diabetes induces its own distinct features of neuropathology different from AD. There are common pathological features to be considered including vascular abnormalities, a major feature arising from diabetes; there is increasing evidence that vascular abnormalities can contribute to AD. The most important common mechanism between insulin-resistant (type II) diabetes and AD could be impaired insulin signaling; a form of toxic amyloid can damage neuronal insulin receptors and affect insulin signaling and cell survival. It has even been suggested that AD could be considered as “type 3 diabetes” since insulin can be produced in brain. Another common feature of diabetes and AD are increased advanced glycation endproduct-modified proteins are found in diabetes and in the AD brain; the receptor for advanced glycation endproducts plays a prominent role in both diseases. In addition, a major role for insulin degrading enzyme in the degradation of Aβ peptide has been identified. Although clinical trials of certain types of diabetic medications for treatment of AD have been conducted, further understanding the common pathological processes of diabetes and AD are needed to determine whether these diseases share common therapeutic targets.

## 1 INTRODUCTION

Alzheimer’s disease (AD) is the most common cause of dementia in the elderly resulting in significant morbidity and health care costs. Depending on populations being studied, the incidence of AD is 10% in those over 65 years of old, rising to 50% in those over 85 years of age. By the middle of this century, as the population progressively ages, AD is becoming an epidemic that will affect 13.2 to 16 million in the United States [1, 2]. Current approved therapies involve the use of acetycholinesterase (AChE) inhibitors that target the symptoms of AD and do little to affect disease progression [3]. Many pathological processes are involved in AD pathology. As this disease can take 15-25 years to manifest as overt cognitive decline, many of these processes are occurring in the brain and periphery simultaneously. Taking into account 25 years of research on AD, it can be concluded that AD results in an accumulation of misfolded proteins, which results in oxidative stress, inflammation and which is contributed to by these factors, leading to mitochondrial damage and synaptic dysfunction. AD is a disease of synaptic failure particularly in the hippocampus.

Although the majority of research in AD has focused on the amyloid beta (Aβ) peptide and how it is generated and metabolized, the role of insulin, glucose regulation and diabetes has been a constant area of research. Glucose metabolism has a pivotal role in maintaining all aspects of the health of the brain; glucose dysregulation has a multitude of pathological effects with many of these being observed in patients with diabetes. It has been known for many years that reduced glucose metabolism, as demonstrated by positron emission tomography using Fluorodeoxyglucose as ligand, is an early indication of the development of AD [4-6]. The significance of these scans is that as changes can be identified before significant neuronal loss, the reduced glucose metabolism is a strong indication of insulin signaling defects in brain being an early pathological event. In addition, intranasal administration of insulin to ensure cerebral rather than peripheral uptake is an effective method of enhancing cognition, both in animal models and now AD patients [7]. The major mystery is that the brains of diabetics do not have enhanced AD pathology [8] so the link between cause of insulin deficiency and AD is still unclear.

Insulin is the major hormone that regulates glucose metabolism in the periphery and the brain, so it can be expected that diseases with glucose dysregulation, namely Type I and Type II diabetes, will also have consequences on brain function. There has been a large body of basic scientific and epidemiological studies showing how diabetes can accelerate AD. Based on these findings one might expect that treating diabetes will slow down or reverse AD neuropathological changes. There have been many excellent reviews over the last 5 years that make this case in a compelling manner; however the aim of this review is to consider this question from a human pathological point of view. *Why are there not enhanced AD pathological changes in the brains of elderly with diabetes?* Does this mean the two diseases have no interrelationship or are the pathological mechanisms of diabetes and AD only sharing some of the features? We will examine many of the common features of diabetes and AD to determine how they might interact and also address the issue of using diabetic medication and/or insulin as AD therapy.

In Fig. (**1**), a simplified scheme of the interaction between insulin deficiency/resistance (not necessarily diabetes) is proposed. As we will examine, some of the features of how failure of insulin signaling leads to AD pathology has been better elucidated in animal models rather than in humans.

### 1.1 Glucose Metabolism as a Biomarker for AD

Positron emission tomography (PET) using radiolabeled fluorodeoxyglucose (FDG) has become one of the oldest and most accurate biomarker tools for identification of mild cognitive impairment (MCI) and early stage AD in living subjects [9-12]. Results show that there is significant regional cerebral glucose hypometabolism early in the disease. These findings have been replicated over approximately the last 20 years and are now being validated using new amyloid imaging ligands [13]. FDG-PET measurements have been shown to correlate well with transition form MCI to AD, and with biochemical biomarkers of early AD, including CSF Aβ and tau levels [14-17]. As these changes in glucose metabolism are occurring early in the disease, it can be suggested that these changes are likely due to defects in insulin signaling, not due to neuronal cell loss. One study showed that structural gray matter alterations were detectable in mild AD in amygdala and hippocampus, while changes in glucose metabolism were occurring in posterior cingulated gyrus and parietal lobes [18]. It was shown in a volunteer population using FDG-PET that administration of insulin resulted in significant enhanced cortical glucose metabolism [19]. This effect was least in cerebellum, a brain region not significantly affected in AD. Reduced FDG-PET uptake was observed in a population with mild hyperglycemia; the reduction in glucose metabolism was in similar brain regions affected by AD [20]. 

### 1.2 Diabetes and AD

Diabetics develop a diverse range of pathological conditions, both in brain and periphery, due to the consequences of glucose dysregulation; as many of the pathologies share features with AD or putative AD processes, research has hypothesized that diabetes (Type I or Type II) is a risk factor for AD. Insulin, a polypeptide hormone consisting of a 21 amino acid α chain and a 30 amino acid β chain, plays the central role in regulating energy metabolism in the body. Diabetes as the “sugar” disease has been identified for centuries, but it has been shown that insulin has numerous neurotrophic, metabolic and endocrine functions in the brain, being centrally involved in memory and neuronal survival (reviewed in [21,22]). This hormone is mainly produced by the beta cells of the Islets of Langerhans of the pancreas, but expression of insulin mRNA in brain has been detected [23]. Type-1 diabetes (T1D) develops when insulin synthesis declines after the Islet cells are lost, potentially due to autoimmune causes or viral infections. T1D, also called insulin-dependent diabetes, is treated by administration of insulin through injection. Without these injections, this disease is invariably fatal due to the subject not being able to maintain adequate glucose levels. 

### 1.3 AD and Type-2 Diabetes

Type-2 diabetes (T2D) also referred to as insulin-resistant diabetes or non-insulin dependent diabetes, tends to be an age-related disease frequently associated with obesity, smoking, and failure to exercise, and characterized by endogenous insulin resistance. Subjects have hyperinsulinemia, hyperglycemia, glucose intolerance, atherosclerosis, hypertension and adiposity [24], which are all risk factors for AD. The cause of T2D is not fully understood, but a range of experimental studies have associated insulin resistance with reduced levels of insulin receptor expression, reduced insulin receptor kinase activation and reduced activation of insulin receptor substrate (IRS)-1 and downstream signaling, and increased expression of inflammatory associated proteins [25-28].

Clinical studies have examined the shared risk for AD and T2D. It has been observed that 81% of AD patients had either T2D or impaired fasting glucose levels [29]. These studies raised an interesting question whether AD patients were predisposed to T2D, or if T2D accelerated AD. It has been consistently observed in epidemiological studies that diabetes, particularly T2D, is associated with cognitive decline; these studies are the foundation of the hypothesis that diabetes could be accelerating pathological processes in AD [30-33]. The potential role of insulin and/or diabetes in precipitating or accelerating AD has been reviewed extensively (representative examples [21, 30, 34-39]). Many have taken the position that there is a positive association between diabetes or insulin signaling deficits and AD based on epidemiological data as well as animal or cellular models. However, it is our supposition that the data have been unclear and conflicting, and now is not supported by neuropathological examination of brains from AD patients with diabetes [40]. If diabetes does not lead to increased AD pathology, one could suggest that diabetic medications will not be effective in slowing AD pathology [41].

## 2 FEATURES OF AD AND INSULIN DEFICIENCY

AD is the most common cause of dementia in the elderly whose incidence is increasing as the population is becoming older. The disease manifests as short-term memory loss that progresses in severity to global dementia. Usually the cognitive decline in AD is associated with the progressive development of extracellular senile plaques, which consist of fibrillar aggregates of amyloid-β (Aβ) deposits, and intracellular neurofibrillary tangles (NFT), composed of hyperphosphorylated tau protein located in the cortex and hippocampus of the affected brain; the disruption of synaptic function; and cerebral atrophy. The central issue for understanding the interaction of diabetes and AD has been to determine if and how diabetes/insulin deficiency can accelerate these above listed pathological features. This has brought up a number of potential mechanisms, including the interaction of insulin degrading enzyme (IDE) in AD and diabetes [42-44]; the involvement of advanced glycation endproduct-modified proteins and the receptor for advanced glycation endproducts (RAGE) [45-47], the interference of Aβ with insulin receptor signaling [39,48-52]; and the role of insulin sensitizers in neuroprotection [53,54].

### 2.1 Insulin and Insulin Growth Factor in the Brain

Insulin and insulin-like growth factor (IGF) have been proven to have vital roles in the brain. They control the neuronal functions by directing the metabolic, neurotrophic, neuromodulatory and neuroendocrine actions that are necessary elements for learning and establishing memory [34]. Studies showed that IGF receptors are widely distributed on neurons and astrocytes [55, 56], and insulin receptors (IR) are expressed in the cerebral vasculature and can mediate the transport of insulin across the blood brain barrier (BBB) [56]. Insulin and IGF immunoreactivity has been observed in human brain, and can be localized to neuronal structures by immunocytochemistry [57]. This could still arise due to neuronal uptake of insulin transported into the brain. However, insulin, IGF-I and IGF -II mRNA have been detected in human brain samples indicating cerebral synthesis occurs. Levels of insulin mRNA were significantly lower in AD samples compared to matched control samples; however, the expression of insulin growth factors (IGF-I or IGF-II) and insulin receptor (IR) mRNA levels were similar between AD and control samples. By comparison, earlier studies using immunohistochemistry showed increased insulin and insulin receptor immunoreactivity in AD and Parkinson’s disease (PD) tissue sections [57-59]. It is now generally agreed that there is a deficit in insulin and IGF-1 signaling in AD brains, which leads to the question that has been hard to resolve: *whether there is an insulin deficit or insulin resistance in the brain*. Additionally, insulin and IGF-I have proved to have direct effects on AD pathogenic processes. They affect the metabolism and clearance of Aβ, and reduce the formation of neurofibrillary tangles by regulating the levels of phosphorylated tau protein. This, in theory could give them potent neuroprotective characteristics [60, 61].

### 2.2 Insulin Production in the Central Nervous System

The majority of the body’s insulin is produced by the pancreas; however insulin can cross the BBB from the circulation to the brain by a receptor-dependent mechanism [43, 62, 63]. As levels of expression of insulin in brain are modest compared to circulating levels, it is still believed that transport of peripheral insulin across the BBB and the consequences of peripheral hyperinsulinemia or hypoinsulinemia have significant roles in cerebral insulin signaling. Insulin binding activity has been identified in the brain in a number of species, including humans [64-66]. However, recent work by De La Monte and colleagues has supported that insulin produced in the brain plays a central role in insulin signaling in the central nervous system [23,67,68]. The two main insulin actions in the brain are regulation of glucose intake and indirectly effects on cognitive functions. In an insulin resistance condition, there is a reduced sensitivity to insulin, which leads to overproduction of insulin (hyperinsulinemia). Chronic peripheral hyperinsulinemia results in an increase of insulin in the cerebrospinal fluid (CSF) but reduced insulin signaling in brain [69].

### 2.3 Diabetes and AD – Pathology Studies

It is incontrovertible that diabetes, particularly T2D, and AD are coexistent diseases of the elderly. The association between diabetes mellitus, insulin resistance and the degree of hippocampal and amygdalar atrophy was investigated in Rotterdam study by volumetric magnetic resonance imaging [70]. In this population-based cohort study, subjects with diabetes mellitus showed more hippocampal and amygdalar atrophy compared to subjects without diabetes. Furthermore, increasing insulin resistance was associated with more amygdalar atrophy. T2D was associated with smaller hippocampal and amygdalar volume regardless of the degree of vascular pathology [70].

In general, diabetes leads to its own type of neuropathology, cerebral neuropathology of type-2 diabetes, which consists of cerebrovascular and white matter pathology [71-73]. In this study, when postmortem pathological assessments were carried out, neuropathological analyses generally demonstrated reduced levels of AD pathology (numbers of amyloid plaques and neurofibrillary tangles). This has now been reproduced in separate studies [8, 71, 74, 75]. Nelson and colleagues examined a large series of elderly diabetic and non-diabetic brains for the signature neuropathology of diabetes [71]. Diabetics had significantly more infarcts, indicative of vascular damage, while these cases had significantly lower plaque scores, as measured by Consortium to Establish a Registry for Alzheimer Disease (CERAD) criteria, fewer tangles in the subiculum, and fewer neuritic plaques in the temporal lobe. Other brain regions had fewer tangles and plaques in diabetic cases, but these differences did not reach statistical significance [71]. A similar study examined for AD pathology (neuritic plaques and neurofibrillary tangles) in diabetics that were treated and untreated, and compared the results with non-diabetics [74]. Similar to the results of Nelson [71], less AD pathology was present in diabetics. The difference was most noticeable in diabetics that had received insulin and insulin combined with an oral anti-diabetic medication. A comparison of diabetic and non-diabetic subjects for the severity of neuritic plaques and neurofibrillary tangles in the cerebral cortex and in the hippocampus found significantly fewer neuritic plaques and neurofibrillary tangles in the cerebral cortex of those with T2D compared to non-diabetics. In the hippocampus, diabetics had significantly lower plaque scores than non-diabetics, but the lower ratings of neurofibrillary tangles did not achieve statistical significance. This study did not show increased AD pathology in subjects with an apolipoprotein E epsilon-4 allele (apoE4) genotype and diabetes compared to apoE4 without diabetes.

This raises the question whether diabetes can accelerate AD pathology, or there are common coexisting factors for both diseases. The reduced AD neuropathology in the brains of diabetics may indicate that treatments for diabetes are reversing the AD pathology. This is germane in deciding whether diabetic medications should be tested for treating non-diabetic AD patients. In a further neuropathology study, investigators examined relation of diabetes to the most common neuropathology causes of dementia, cerebral infarction and AD [75]. They found a relationship between diabetes and cerebral infarction but not between diabetes and AD pathology in older persons. Their findings indicate that those with diabetes are more likely to acquire enhanced levels of cerebrovascular disease, but not differing amounts of AD pathology compared to those who do not have diabetes [75]. At present, the data does not support the hypothesis that diabetes is a direct causal factor for AD. However, we will review the evidence that a defect in insulin and insulin signaling in the brain is a significant co-factor for AD.

### 2.4 Interactions of Diabetes, Apolipoprotein E4 and AD

Vascular abnormalities including atherosclerosis are increased in diabetics, and also are a feature of those possessing the apoE4 allele. Possession of a single or two copies of the apoE4 allele is the most significant risk factor for developing sporadic AD [76, 77]. Subjects with at least one apoE4 allele tend to have higher levels of atherosclerosis and vascular amyloid than apoE4 negative subjects [78, 79]. It has been suggested that there might be an interaction of diabetes and AD with apoE4, though studies have produced conflicting data. In an AD population, diabetes had 13% incidence in a non-apoE4 population, and a 5% incidence in AD subjects with one apoE4 allele [80]. However, individuals with T2DM who possess an apoE4 allele have twice the risk of developing AD compared with non-diabetics subjects with an apoE4 allele [81].

In a study of an Italian population aimed at the diagnosis and treatment of dementia, vascular factors such as hypertension and hypercholesterolemia were not found to be significantly associated with disease progression, but, surprisingly, patients with diabetes had a 65% reduced risk of fast cognitive decline compared to AD patients without diabetes. Their findings suggest a slower disease progression in AD patients with diabetes, when based on clinical progression of AD severity [82]. As most of the patients were being treated for diabetes, this again could suggest an effect of diabetic medication/treatment being beneficial for AD*.*

### 2.5 Diabetes, AD and Vascular Dementia

We are still left with the conflicting situation that epidemiological studies show increased frequency of dementia in diabetics, while the series of neuropathology studies did not show enhanced AD pathology in this same group [83-88]. The Rotterdam Study demonstrated a significantly higher incidence of dementia in patients with both insulin dependent (T1D) diabetes and T2D compared to age-matched controls [24, 89, 90]. These studies suggested diabetes doubled the risk of dementia. Interestingly, one of these studies demonstrated a greater risk of dementia in subjects being treated with insulin [90]. The risk of both AD (with Relative Risk RR 1·5–27·0) and vascular dementia (RR 2·0–2·5) was found to be increased in subjects with T2D [91]. Some epidemiological data suggest that enhanced incidence of AD in diabetes may actually be due to vascular forms of dementia. Studies showed that there is a cluster of risk factors for T2D and vascular diseases, which include high blood glucose, obesity, hypertension, increased blood triglycerides and insulin resistance. All of these factors, both individually and collectively, increase the risk not only of AD, but also vascular dementia [92, 93].

### 2.6 Hypertension is a Common Feature of T2D

The effect of antihypertensive drug use on dementia in a cohort of the population-based Rotterdam Study was investigated [94]. They demonstrated that subjects taking antihypertensive medication had a reduced incidence of dementia. This reduction risk was most significant for vascular dementia (VaD), not for AD. Both cerebrovascular disease and T2D decrease cognitive functioning in elderly people, but it is uncertain if T2D affects cognition independent of vascular disease. Patients with arterial disease and T2D performed worse on neuropsychological tests compared to similar patients without T2D [33]. Elevated systolic and diastolic blood pressures were significantly associated with decreased cognition in patients with T2D. Large infarcts and global and cortical atrophy on MRI were independently associated with cognition in patients with T2D. They concluded that the presence of T2D in patients with symptomatic arterial disease is associated with decreased cognitive functioning. Cerebrovascular disease and AD are common diseases of aging and frequently coexist in the same brain.

## 3 BIOCHEMICAL MECHANISMS LINKING INSULIN AND AD

### 3.1 RAGE, Diabetes and AD

A common pathological feature between diabetes and AD has been the presence of advanced glycation endproduct (AGE)-modified proteins in both diseases [47, 95]. These modified proteins are ligands for the receptor for advanced glycation endproducts (RAGE), as is Aβ peptide [96]. RAGE, was first recognized as a cell surface receptor for the products of non-enzymatic glycation and oxidation of proteins, being involved in many inflammatory process, and is a component of major histocompatibility complex (MHC) gene cluster [97]. RAGE expression is influenced by pathological conditions such as diabetic vascular disease, chronic inflammation and AD [98, 99], as its interaction with AGE-modified proteins in either diabetes or AD, or Aβ in AD, has the potential to produce damaging inflammatory responses [100, 101]. In multiple studies of diabetes or AD models, it has been shown that increased presence of RAGE ligands leads to pro-inflammatory activation by RAGE-expressing cells; also with the consequence of increasing amounts of RAGE being expressed. Furthermore, this interaction between RAGE and its ligands triggers the activation of a key cell signaling pathway, such as p21^ras ^and transcription factor nuclear factor-kappa B (NF-κB). 

The activation of NF-κB is considered to be the most common feature in RAGE signaling, as it up regulates cellular expression of RAGE [100, 101]. The extent that this happens *in vivo* is still unclear, but increased RAGE expression along with increased amounts of AGE-modified proteins is believed to be a major cause of the vascular complications that are a common feature of diabetes and AD [102-107]. In diabetes, the elevated blood glucose level promotes formation of advanced glycation end products (AGEs). Continuous hyperglycemia is a causative factor for diabetic vascular complications primarily due to the enhanced generation of advanced glycation end-products (AGEs). Different types of AGE-modifications can occur; recent evidence suggests that glyceraldehyde-derived AGEs (glycer-AGE) are the predominant modification of the most toxic forms of AGEs (TAGE). Glycer-AGE-modified proteins are directly toxic to cultured neurons; diabetic serum enriched in glycer-AGE modified proteins was also toxic to neurons, an effect that could be neutralized with antibody to glycer-AGE. Moreover, increased amounts of these modified proteins were detectable by immunocytochemistry in AD hippocampus tissue sections [95, 108-110]. On the other hand, immunohistochemistry for RAGE in AD brains has been demonstrated with increased expression in neurons, microglia, astrocytes and vascular endothelial cells [96, 111-114]. It has been shown that RAGE is a receptor for Aβ [96], and it mediates the transport of plasma Aβ across the BBB [115], supported by the evidence that the blockade of RAGE on brain vascular endothelial cells reduce transport of peripheral circulating Aβ into the brain [92]. In addition, the migration of monocytes across the human brain endothelial cells in response to Aβ was mediated by RAGE [116].

### 3.2 Insulin Signaling and Aβ

As studies have concluded that diabetic neuropathology and AD are separate entities, the mechanism of insulin’s involvement in AD needs to be resolved. As mentioned, up to 80% of AD patients have type II insulin resistance diabetes (T2D). In T2D, there is an excess production of insulin due to the insulin receptors becoming unresponsive to insulin binding. The increased production of insulin can compensate for the lack of signaling to some extent, but this excess insulin is degraded by the pool of IDE, which also functions to degrade monomeric Aβ. This results in increased levels of both, and the resulting lack of insulin signaling. Blocking insulin signaling results in reduced glucose metabolism, increased glycogen synthase kinase-3β (GSK-3β) activation which can promote hypophosphorylation of tau and increased Aβ production. It has even been suggested that insulin can promote Aβ aggregation. The possible interactions of insulin and Aβ on insulin signaling are shown in Fig. (**2**). The effects of excess type Aβ is generated as a result of proteolytic cleavage of amyloid precursor protein (APP) and has been considered the main toxic mediating factor in AD. It has been shown that Aβ binds to a number of neuronal or glial receptors (reviewed [117, 118]), including the insulin receptor. It appears that monomeric Aβ can compete with insulin for the neuronal insulin receptor [119], with the consequence of reduced insulin receptor signaling. Recent studies have shown that interactions of the specific soluble toxic forms of Aβ, known as Abeta derived diffusible ligands (ADDL), with neuronal insulin receptors have suggested as a neurotoxic mechanism in AD. Extracellular ADDLs have been shown to bind to and inhibit insulin receptor signaling. Binding of ADDLs to synaptic insulin receptors on cultured neurons resulted in their rapid loss from the plasma membrane and their internalization. Intracellular ADDLs can inhibit phosphoinositide-dependent kinase 1 and Akt1, preventing Akt1-mediated cellular signaling, a pathway central for insulin signaling [120,121]. Moreover, it activates GSK3β, which has many substrates including IRS-1, resulting in impaired insulin signaling. In a study to test the hypothesis that insulin signaling provides a protective mechanism against ADDLs toxicity, it was shown that the neurotoxic effects of ADDLs could be competitively inhibited with insulin, or with suboptimal doses of insulin combined with the insulin receptor sensitizing drug rosiglitozone, by blocking ADDLs binding to synapses [122]. As it is hard to maintain the insulin balance in the brain with aging, since insulin signaling tends to decline, this might be the main risk factor for developing AD. Therefore, new insulin focused therapeutic interventions need to be explored to determine if they can preserve brain function [99]. Features of failed insulin signaling in the brain and the potential AD-related consequences are illustrated in Fig. (**2**). 

### 3.3 Insulin Degrading Enzyme in Diabetes and AD

When considering the potential interactions between diabetes, particularly hyperinsulinemia, and AD, the most convincing evidence has come from a range of studies on insulin degrading enzyme (IDE). IDE also referred to as insulysin or insulin protease, is a zinc binding metalloprotease involved in regulating insulin levels and is one of the proteases involved in Aβ degradation (primarily the monomeric and soluble forms of Aβ) [123-127]. Mutations in the IDE gene that result in reduced activity of the enzyme and lead to diabetes in a rat model of T2DM also result in enhanced cerebral deposition of Aβ [128]. IDE has additional substrates including insulin-like growth factor (IGF-II), amylin, glucagon, transforming growth factor alpha and beta endorphin, which will affect its affinity for insulin or Aβ [129]. The hypothesis of the interactions of IDE, insulin and Aβ/AD has come from studies on T2D. In such a condition with hyperinsulinemia, where cells are not sensitive to insulin regulation, the extra insulin produced competes for the IDE and lowers the amount available for Aβ degradation [130]. This mechanism has been demonstrated in a transgenic model of insulin resistant diabetes [131]. Hence, this could be a mechanism for increased build up of Aβ, the most established pathological feature of AD. There is an interesting interaction between insulin, IDE and cholesterol metabolism. Mice fed a high fat diet had reduced IDE mRNA and protein expression and elevated Aβ levels, while in humans, lowest levels of IDE (and neprilysin, the other major Aβ degrading enzyme) were in AD cases that were homozygous for Apo 4 allele [132,133].

### 3.4 Insulin Resistance Syndrome, Memory and Inflammation

Insulin resistance syndrome is a condition in which there is an impaired response of body cells (muscle, fat, and hepatic) to insulin resulting in impaired endothelial function and enhanced atherosclerosis [134-138]; which requires higher doses of insulin for normal function. Insulin is known for its significant role in establishing and preserving brain memory [139]. With aging and other memory impairment diseases (as in AD), insulin levels increase in the plasma, while it tends to decrease in the CSF and has reduced activity in the brain [140] . Insulin resistance and hyperinsulinemia have proved to be risk factors for both general memory decline and AD [87,141]. Furthermore, studies have revealed that during a chronic insulin resistance state or chronic inflammation, insulin may aggravate inflammatory responses, increase markers of oxidative stress and lead to the formation of superoxide anions [141-143]. The administration of insulin has resulted in increased plasma concentrations of C-reactive protein and pro-inflammatory cytokines (IL-1β, IL-6, and tumor necrosis factor α [TNFα] [144].

### 3.5 Glucose, Insulin and Memory

The human brain uses up to 25% of total body glucose, as it is the main nutrient for maintaining brain function [145]. However, hyperglycemia leads to many pathological changes. One biomarker for studying hyperglycemia in living patients is serum glycohemoglobin (HbA1C) [146-148]. These studies were carried out in different populations, but showed that uncontrolled diabetes/hyperglycemia as shown by elevated HbA1C correlated significantly with declines in cognitive function. As reviewed, the mechanisms that mediate the toxic effects of hyperglycemia are varied, but include the accumulation of AGEs; increases in reactive oxygen species (ROS) production; enhanced vascular inflammation leading to microvascular changes that can result in microinfarcts and generalized brain atrophy. This supports the hypothesis that glycemic control is the cornerstone in maintaining functional memory in patients with T2D [149].

### 3.6 Animal Models for CNS Insulin Signaling Deficits in AD

One of the problems of human clinical studies of diabetes and AD has been the fact that measurements of glucose metabolism and insulin levels at the time of death in an AD patient are not reflective of the pathological mechanisms that lead to this stage. Most of the metabolic processes will have been altered prior to death. For this reason, relevant animal models have been invaluable for dissecting insulin related mechanisms in the CNS and whether they predispose to AD.

A widely used reagent for inducing experimental diabetes is streptozotocin, broad spectrum antibiotics with a nitrosamine-related compound that destroys beta cells in the pancreatic islets. Injection of streptozotocin into the ventricles of the brains of rats resulted in 10-30% reduction in glucose metabolism. The mechanism of this cytotoxic diabetogenic drug is mainly mediated by the development of ROS, in addition to the formation of nitric oxide (NO), which induces destruction by necrosis [150]. It is presumed that the same mechanism occuring in the pancreas occurs in the brain. The widely used transgenic Aβ plaque-developing mouse models of AD have been used in a number of studies to demonstrate how insulin resistance or deficiency can accelerate plaque deposition and memory impairment. The key studies are reviewed below while considering different insulin related mechanisms. However, the interpretation of these findings as they relate to the human diabetic needs to be interpreted carefully before considering the indicated therapeutic approaches. Although widely used, there are limitations to these mouse models of AD as they relate to the human disease.

#### 3.6.1 Transgenic Mice and Insulin Degrading Enzyme

Increased generation of Aβ appears to stimulate expression of IDE in AD transgenic mice model. As plaque formation progresses, IDE levels increase. This was particularly noticeable with the increased numbers of IDE-immunoreactive astrocytes surrounding plaques [151]. These investigators showed that Aβ could directly stimulate IDE expression by astrocytes *in vitro*. This finding contrasts with the decrease in IDE shown in human AD brains [132]. This finding was replicated in another study where increased expression of IDE occurred with the progressive increased accumulation of Aβ levels in a transgenic mouse model [152]. Of more relevance, using Tg2576 mice that had been fed a diet that induces type-2 like insulin resistance, these animals had enhanced levels of Aβ compared to control animals, and this treatment also resulted in lower levels of IDE [131].

#### 3.6.2 Transgenic Mice and Insulin Signaling

Studies of insulin deficiency, as a model of type 1 diabetes, in transgenic mice provided some parallel features with AD. Nine weeks of insulin deficiency resulted in reduced IDE, reduced learning capacity, and reduced insulin receptor signaling. Reduced insulin signaling resulted in increased tau phosphorylation and Aβ levels. These effects were partially reversed with insulin treatment. These pathological features were not observed in a parallel T2D model [153]. Two new transgenic mouse models have been produced that demonstrate how AD pathology can exacerbate insulin signaling deficiencies and glucose tolerance and how diabetes can enhance cerebrovascular Aβ deposition and amyloid mediated vascular inflammation. Crossing APP23 transgenic mice with *ob/ob* (leptin deficient) mice resulted in enhanced diabetes symptoms (blood glucose levels and plasma insulin) compared to *ob/ob* mice alone, but with reduced insulin levels in the brain. These mice also had significantly reduced cognitive function and insulin signaling, even though there was no increased Aβ compared to APP non-diabetic mice. Similarly, crossing APP23 with the mouse model of T2D (Nagoya-Shibata-Yasuda (NSY)) resulted in more severe memory deficits and insulin resistance compared to NSY mice. In this model, brain Aβ levels correlated positively with circulating glucose levels. This study concluded that the enhancement of Aβ-associated memory impairment and pathology in these mouse models was due to increased cerebrovascular inflammation mediated by increased RAGE expression on endothelial cells. It still remains to be determined how vascular inflammation can affect insulin signaling, but this and other studies suggest that it can reduce BBB transport of insulin into brain [154]. Two recent studies investigating the effect of T1D on amyloid plaque developing transgenic mice came to similar conclusions that diabetes/insulin deficiency was exacerbating the AD-like pathology [155, 156]. Both induced T1D by injections of streptozootocin, one by intraperitoneal injection [156], and the other by intracerebral injection [155]. Both studies showed reduction of cognitive measures and enhanced levels of phosphorylated tau and Aβ plaques. Similarly, using a different genetic mouse model that develops tangles, not plaques, due the presence of the P301L tau mutation, streptozootocin-induced T1D accelerated the formation of phosphorylated tau and tangle formation [157]. At present, it is hard to determine which mouse model of the interaction of insulin signaling defects and AD best reflects the human condition. As discussed, diabetes in humans does not appear to enhance AD neuropathology. In this final section, we will discuss how insulin focused therapy might be effective in treating some of the clinical manifestations of AD. 

## 4 NEUROPHARMACOLOGY OF INSULIN THERAPY

The literature on diabetes, dementia and therapy has drawn many divergent conclusions possibly due to the differing designs of the clinical studies. To address the issue whether insulin therapy or anti-diabetic medication could affect cognitive function, a group of AD patients with T2D were either treated with anti-diabetic medication, or with insulin and anti-diabetic medication. After 12 months, the group receiving insulin and anti-diabetic medication had deteriorated at half the rate of those receiving the non-insulin therapy [158]. Another recent study that showed that the rate of cognitive decline in AD patients with diabetes was significantly less than those without appeared to conflict with the previous study, however the patients with diabetes were being treated with insulin and/or anti-diabetic medication (though the percentages were not reported) [159], so the findings of both papers that diabetic/insulin medication is useful for treating AD cognitive decline may be similar.

### 4.1 Current Treatment Plans for DM and AD

The treatment strategies that are now used in AD are mainly targeting symptom improvement. AD has a major inflammatory component due to the neurodegeneration and Aβ peptide deposition. As a result, many studies have shown that non-steroidal anti inflammatory drugs (NSAIDs) may affect AD process, and may reduce the risk and delay the onset of AD. Some NSAIDs are also ligands for peroxisome proliferator activated receptors (especially the PPARγ) [54, 132, 160, 161].

PPARs are ligand-inducible transcription factors that regulate the expression of a number of inflammatory and insulin-regulated genes [160]. The best-known PPAR ligands are the thiazolidinediones such as rosiglitazone and pioglitazone, which are insulin sensitizers and are used in the treatment of T2D. Recent studies have shown that pharmacological treatment with thiazolidinediones may offer some therapeutic answers for AD by lowering peripheral insulin and improving insulin sensitivity [162-165]. Furthermore, these agonists of the nuclear receptor have also been shown to reduce Aβ accumulation, inflammatory reactants and exhibit neuroprotective effects in animal models [163, 164, 166]. In a prospective randomized, open-controlled study, 32 diabetic patients with mild to moderate AD were enrolled. Subjects treated with insulin were excluded. Patients were randomly assigned to the treatment with pioglitazone, as they were not taking any medication known to affect cognition or behavior during the study. Results showed significant cognitive and metabolic improvements in diabetic patients with AD after 6 months of taking pioglitazone compared with a control group [162]. Another randomized study in mild to moderate AD patients was conducted using rosiglitazone [167]. Results were stratified by apoE genotype. This study detected no significant statistical differences between placebo and any rosiglitazone (RSG) dose at baseline. However, there was a significant interaction between apoE4 allele status and Assessment Scale-Cognitive (ADAS-Cog). It showed a significant improvement in ADAS-Cog in apoE4-negative patients on a high dose of RSG (P=0.024) while apoE4-positive patients showed a decline at the lowest RSG dose (P=0.012). These results showed that apoE4 non-carriers might be the only subjects that would have cognitive and functional improvement in response to RSG [167].

Recent findings on the safety of rosiglitazone might preclude this drug for further testing in AD, but alternative drugs of this family with similar modes of action are available.

### 4.2 Effects of Intranasal Administration of Insulin on Cognition and AD 

Another new approach in treating AD is in the administration of intranasal insulin. The fact that there are direct pathways between the nasal cavity and the central nervous system has guaranteed an easy access for insulin through the BBB, without the risk of systemic hypoglycemia that results from the peripheral administration of insulin [7]. In this study, insulin levels increased in the CSF 10 minutes after the intranasal administration with peak levels in 30 minutes [7]. Recent studies were conducted on patients with mild cognitive impairment and AD using intranasal insulin. Findings have shown that the administration of intranasal insulin in average dose of 20 IU acutely benefits the verbal memory in non apoE4 carriers. On the contrary, to relative decline or no benefit in apoE4 carriers. These findings suggest that dose-response is different by apoE4 genotypes, which could be due to insulin sensitivity differences that are common in non apoE4 AD patients [7]. 

## CONCLUSION

Initially, insulin was thought to not cross the BBB as the brain was perceived to be an insulin–insensitive organ, however this was shown not to be true. It is still probable that most insulin in the brain is transported from the periphery but further studies on CNS synthesized insulin are needed [168]. Insulin has proved to be a crucial element for neurological functions. T2D and chronic peripheral hyperinsulinemia are associated with impairment in memory and cognitive functions. The exact pathophysiology of cognitive dysfunction and cerebral lesions in diabetes mellitus is not completely understood. However, hyperglycemia, vascular disease, and insulin resistance are considered to be the main factors [169]. Diabetes and AD appear to have common features, and some authors refer to AD as type 3 diabetes. The factors that support the relationship between T2D and AD are insulin resistance, altered brain IDE levels, Aβ clearance and AGEs formation. Treatment strategies for both diseases have been considered, because of the shared features. Diabetic medications have been explored as treatment candidates for AD. Insulin-sensitizing agents (ligands for PPARγ) and intranasal insulin have been the most recent candidates, though insulin-sensitizing agents now appear to have limited effects and significant side effects. However, they have offered a potential but limited therapeutic solution, and more research on patients with both diseases is needed to answer the questions being proposed on insulin and AD.

## Figures and Tables

**Fig. (1):**
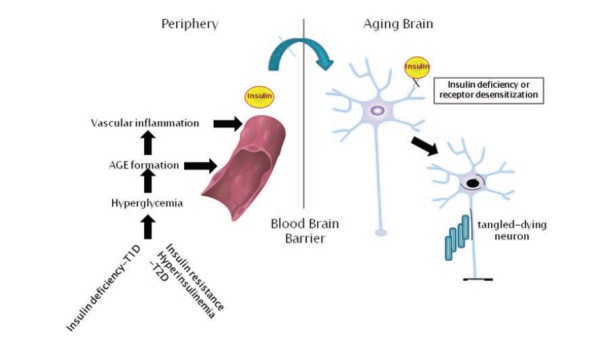
**Potential periphery to brain links between type 1 and type 2 diabetes and Alzheimer’s disease.** Peripheral insulin deficiency or insulin resistance leading to hyperglycemia, increased deposition of advanced glycation endproduct (AGE) modified proteins in the vasculature and increased vascular inflammation. These events lead to reduced transport of insulin across the blood brain barrier to the brain. Reduced neuronal insulin receptor signaling, due to insulin deficiency or insulin receptor desensitization alter a number of cellular signaling pathways that can promote amyloid beta (Aβ) formation, phosphorylation of tau and reduced glucose uptake and metabolism by neurons.

**Fig. (2):**
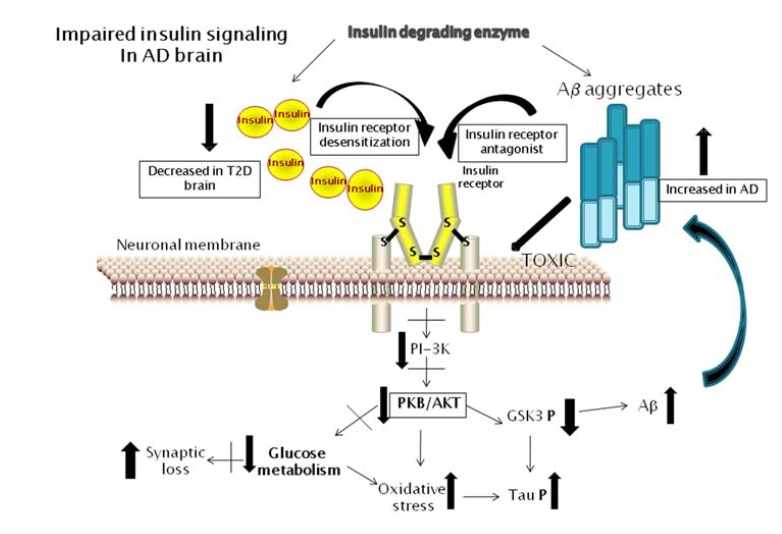
**Consequences of insulin and Aβ interactions on reduced neuronal insulin receptor signaling and promoting Alzheimer’s disease pathology.** In type 2 diabetes, there can be decreased or increased levels of insulin in brain (depending on disease state), along with insulin receptor desensitization. Aβ peptide levels can be enhanced by reduced insulin receptor signaling, and soluble Aβ oligomers can also block these receptors. Increased Aβ levels will compete for insulin degrading enzyme with cerebral insulin. Aβ aggregates can also have direct membrane toxic effect on neuronal cells. Reduced insulin receptor signaling results in reduced PI-3K activity that leads to reduced PKB/AKT activity. The consequences of this include reduced glucose metabolism and increased oxidative stress. Specifically reduced GSK3 phosphorylation leads to increased tau phosphorylation and Aβ formation. Abbreviations: PI- 3K- phosphoinositide-3 kinase: PKB/AKT – protein kinase B; GSK3 – glycogen synthase kinase 3: Aβ - amyloid beta peptide.

## ABBREVIATIONS

Aβ: = Amyloid beta peptide
AChE: = Acetylcholinesterase
AD: = Alzheimer’s Disease
ADDL: = Abeta Derived Diffusible Ligands
BBB: = Blood Brain Barrier
CSF: = Cerebrospinal Fluid
FDG: = Fluorodeoxyglucose
HbAIC: = Glycohemoglobin
IGF: = Insulin-Like Growth Factor
IDE: = Insulin Degrading Enzyme
NO: = Nitric Oxide
PD: = Parkinson Disease
PET: = Positron Emission Tomography
PPAR: = Peroxisome Proliferator-Actived Receptor
RAGE: = Receptor for advanced glycation endproducts
ROS: = Reactive Oxygen Species
T1D: = Type 1 Diabetes
T2D: = Type 2 Diabetes
